# Structural and Functional Measures of Efficacy in Response to Bevacizumab Monotherapy in Diabetic Macular Oedema: Exploratory Analyses of the BOLT Study (Report 4)

**DOI:** 10.1371/journal.pone.0072755

**Published:** 2013-08-27

**Authors:** Sobha Sivaprasad, Roxanne Crosby-Nwaobi, Simona Esposti, Tunde Peto, Ranjan Rajendram, Michel Michaelides, Philip Hykin

**Affiliations:** 1 NIHR Moorfields Biomedical Research Centre, Moorfields Eye Hospital, London, United Kingdom; 2 UCL Institute of Ophthalmology, University College London, London, United Kingdom; Fundación para la Prevención y el Control de las Enfermedades Crónicas No Transmisibles en América Latina (FunPRECAL), Argentina

## Abstract

**Background:**

To describe structural and functional changes associated with diabetic macular oedema (DMO) treated with intravitreal bevacizumab over 24 months.

**Methods:**

A post-hoc analysis of the data of 34 patients that completed 24 months follow-up in the intravitreal bevacizumab arm of a prospective, randomized controlled trial (BOLT study) was performed. The outcome measures previously used in clinical trials of intravitreal ranibizumab in DMO were employed to describe the visual acuity and macular thickness changes at 12 and 24 months.

**Results:**

The standard outcomes of mean change in best corrected visual acuity (BCVA) and central macular thickness (CMT) in participants treated with bevacizumab were comparable to those reported in association with ranibizumab. However, exploratory analyses showed that thick maculae at baseline defined as CMT of ≥400 µm, remained significantly thicker than those <400 µm with intensive bevacizumab therapy, despite a comparable gain in visual acuity at both 12 and 24 months. The proportion of subjects that attained a dry macula doubled in both CMT groups between the 12 and 24-month time-points.

**Conclusions:**

These findings provide valuable information both for clinical practice and trials. Further studies are required to investigate the impact of intravitreal bevacizumab on retinal thickness profiles in DMO.

## Introduction

Diabetic macular oedema (DMO) is one of the leading causes of visual impairment in the working age group [1]. Laser photocoagulation has been the mainstay of treatment of this condition [2]. The introduction of molecular inhibitors of vascular endothelial growth factor (VEGF), such as ranibizumab and bevacizumab, as approved and off-label therapy for DMO respectively, has revolutionised the management of DMO, with improved visual outcomes compared to laser photocoagulation [3], [4], [5], [6]. Analyses of several well-conducted clinical trials of ranibizumab in DMO have provided insights into outcome measures that are valuable to clinicians, health economists, funders and clinical trialists [7], [8]. For example, the stratification of DMO and response to treatment in terms of macular thickness has helped to improve our understanding of the effect of ranibizumab.

In comparison, there are fewer randomised clinical trials of bevacizumab in DMO, with smaller patient numbers. Moreover, the inclusion criteria and outcome measures used in these trials are not comparable enough to readily allow stakeholders to reach an informed decision about its use [9]. To begin to address this we have undertaken a detailed analysis of the results of our clinical trial on bevacizumab for DMO (BOLT study) using the accepted outcome measures and predictive factors that have been published in the ranibizumab studies [4], [8], [10], [11].

The objectives of the study were to conduct exploratory analyses to provide further detailed descriptive statistics of the outcome measures of the bevacizumab arm of the BOLT study in line with the DRCR.net study on ranibizumab in DMO [7], [8] and examine the factors that are associated with change in visual acuity and central macular thickness at 24 months.

## Methods

### Ethics Statement

The BOLT study complied with the Declaration of Helsinki. The research protocol was approved by the Moorfields and Whittington Ethics Committee 07/Q0504/28.

### Synopsis of the BOLT Study

The details of the BOLT study and the results based on the standard outcome measures at 12 and 24 months have previously been reported [10], [11]. In brief, the BOLT study was a prospective randomised 2-year single-centre trial that enrolled patients with persistent centre-involving clinically significant DMO after at least 1 prior macular laser treatment (MLT) and compared the efficacy of intravitreal bevacizumab (1.25 mg/50 µl given at 6 weekly intervals) to MLT at 4 monthly intervals. Primary outcome was assessed at 12 months [10] and secondary outcomes at 24 months [11]. Patients with best-corrected visual acuity (BCVA) in the study eye between 35 and 69 ETDRS letters at 4 m (Snellen equivalent ≥6/60 or ≤6/12) and centre-involving DMO with CMT on Stratus optical coherence tomography (OCT) of ≥270 µm were included. In the bevacizumab arm, patients received 3 mandatory injections at 6 weekly intervals followed by an OCT-guided re-treatment protocol. The study reported that a median of nine injections in the first 12 months resulted in a median gain of 8 letters in 42 patients in the bevacizumab arm [10]. Following a median of a further 4 injections in the second year, the median gain of visual acuity from baseline was 9 letters in the 37 patients that continued in the bevacizuamb arm in the second year [11]. In the bevacizumab arm, the median gain of vision was 9 ETDRS letters and 49% gained 10 letters while 32% gained 15 letters at 24 months. The mean reduction in CMT was 146 µm.

### Exploratory Outcome Measures

The functional outcome measures of mean change in BCVA, proportion of patients with gain or loss of visual acuity of 10, 15 and 30 letters at 12 and 24 months have previously been reported [10], [11]. We further evaluated the proportion of patients with >73 letters (Snellen equivalent>6/12) at 12 and 24 months. In addition, the percent reduction in visual acuity deficit was assessed. Visual acuity deficit was defined as 84 letters (Snellen equivalent 6/6) minus baseline visual acuity. The percentage deficit was calculated as the percentage change in visual acuity from baseline divided by the visual acuity deficit [7], [8]. Patients were also stratified into 2 groups based on the UK definition of visual impairment at baseline: <54 letters and ≥54 letters [12], and the above outcome measures were compared in the two groups.

The anatomical change on OCT has been previously reported as the mean change in CMT (central 1000-µm diameter area) as shown on the automatic thickness analysis map [10], [11]. The StratusOCT (Carl Zeiss Meditec, Inc., Dublin, CA) images, using the Radial Lines protocol of six high-resolution B-scans (512 A-scans per 6-mm B-scan) or the Fast Macular Thickness protocol of six lower-resolution B-scans (128 A-scans per 6-mm B-scan) were used. Scans with a signal-to-noise ratio of ≥6 were included. The coefficients of repeatability are less than 8% in healthy subjects and less than 9% in patients with diabetic macular oedema [13]. We further explored the proportion of patients with <250 µm (‘dry macula’) at 12 and 24 months. Trials for diabetic retinopathy have defined CMT values >250 µm as significant for macular edema to qualify for various trials [3], [8], [10]. In order to control for any ceiling or floor effect, the percentage change in CMT was also considered as a treatment response. This was calculated as the change in thickness divided by the baseline thickness.

Excess retinal thickness was defined as baseline CMT minus 201 µm (normative value of CMT). The percentage change in excess retinal thickness was calculated as the percentage of change of CMT from baseline divided by excess retinal thickness [7], [8].

A one log step change in logOCT is 20% change in CMT and this approximates to twice the error of measurement for any degree of retinal thickening on OCT while a 2 log step change is generally considered clinically important at any level of visual acuity [8], [14]. Logarithmic transformation of the OCT retinal thickness helps normalise skewed distribution of these values. This exploratory measure was evaluated at 12 and 24 months in our cohort.

In order to probe the drug-related structure-function relationship, we used the composite score used in the DRCR.net analyses, defined as a 10 letter improvement with at least a 20% reduction in CMT [8]. This was assessed at 12 and 24 months.

In keeping with previously described ranibizumab-associated outcomes, we also classified the anatomical responders as those with evidence of a CMT reduction of ≥20% at 4 and 12 months [8]. In brief, early and consistent responders were those who showed ≥20% reduction in CMT at 4 months and this effect was sustained at 12 months. Early and inconsistent response was defined as those that showed the aforementioned early response but failed to maintain it at 12 months. Slow and variable responders are those who did not respond at 4 months but responded at 12 months. Non-responders are those that did not show any response at 4 months or 12 months [8].

At 24 months, we further classified the anatomical responders based on the change of CMT≥20% from 12 months into three groups: (i) sustained responders were those that showed a further ≥20% reduction in CMT at 24 months; (ii) stable response was defined as a change of CMT of ±19% from 12 months; and (iii) late poor responders were defined as those in whom CMT increased by ≥20% at 24 months.

The participants were also stratified based on baseline CMT into <400 µm or ≥400 µm based on the fact that ranibizumab is most cost-effective when given to patients with CMT≥400 µm than <400 µm [15] and the above outcome measures were compared between these two groups.

### Predictive Factors

The baseline data from the BOLT study that were analysed include age, gender, visual acuity, macular thickness, duration of diabetes, HbA1c (glycosylated haemoglobin), number of previous laser treatments and diameter and area of the foveal avascular zone. The dependent variables were final BCVA, change in BCVA, final CMT and change in CMT.

### Statistical Analysis

Only patients that completed the 24 months follow-up were included in this study. However, baseline characteristics were compared between this group and the whole cohort recruited to the bevacizumab arm of the study. Statistical analysis was conducted utilising association of baseline clinical, OCT, and fundus photographic variables using STATA. All reported *P*-values are 2 sided and a Bonferroni adjustment was made to the multiple comparisons. P-values of less than 0.05 were taken as significant. Statistical significance of sample characteristics were determined using Kruskal-Wallis test for continuous variables and chi-square for categorical variables. Multiple regression analyses, using (General Linear Modelling; multivariate ANCOVA) was used to determine the factors which may be predictive of treatment response.

## Results

A total of 34 patients completed the 24 month exit assessments in the bevacizumab arm of the BOLT study and were included in these analyses. The baseline characteristics of these participants were comparable to those previously reported for all participants that were randomised to the bevacizumab arm [10]. The median visual acuity was 58.5 letters (25^th^ percentile, 53 letters and 75^th^ percentile 64.5 letters). The mean (SD) was 57.6 (7.6) letters. Table 1 shows the BCVA and CMT at baseline, 12 and 24 months. Although all participants in this trial had a baseline BCVA in the study eye that was below the legal requirement to drive in the United Kingdom, treatment with bevacizumab enabled approximately 25% of them to meet the requirement based on final visual acuity being more than 73 letters (Snellen acuity better than 6/12) in the study eye. Similarly, a BCVA of less than 54 letters (Snellen equivalent of 6/24) is the level of vision classified as visual impairment. The proportion of eyes with vision <54 letters reduced by 50% and 60% at 12 months and 24 months respectively.

**Table 1:** The baseline, 12 and 24 months best corrected visual acuity and central macular thickness broken down by categories.

ETDRS letters	Baseline N (%) N = 34	12 months N (%) N = 34	24 months N (%) N = 34
>73	0 (0)	3 (8.8)	9 (26.5)
69–73	3 (8.8)	7 (20.6)	6 (17.6)
59–68	14 (41.2)	15 (44.1)	12 (35.3)
49–58	11 (32.4)	5 (14.7)	2 (5.9)
39–48	6 (17.6)	4 (11.8)	4 (11.8)
≤38	0 (0)	0 (0)	1 (2.9)
**CMT** µ**m**			
<250	0 (0)	6 (17.6)	12 (35.2)
<300	1 (2.9)	11 (32.4)	15 (44.1)
301–400	11 (32.4)	8 (23.5)	7 (20.6)
401–600	17 (50.0)	11 (32.4)	8 (23.5)
>600	5 (14.7)	3 (8.8)	4 (11.8)

ETDRS –early treatment diabetic retinopathy study; CMT- central retinal macular thickness.

The median central subfield thickness was 486.5 µm (25^th^ percentile, 387; 75 percentile, 525). Despite a median of 9 injections in the first year, only 17.6% attained <250 µm at 12 months and this proportion doubled at 24 months (35%). However, the proportion of participants with >600 µm remained unchanged throughout the study period. The mean number of injections given to the patients with a dry macula at 24 months was not significantly different to those that continued to be thickened.

### Visual Acuity Outcome at 12 and 24 Months

The overall mean gain of visual acuity of these participants was 5.2 letters (one ETDRS line of vision) at 12 months and 7.8 letters at 24 months. The changes in various categories of visual acuity are shown in Tables 2 and 3. Participants with baseline BCVA of less than 54 letters gained more letters at 12 months than those with ≥54 letters. The trend continued at 24 months when a mean gain of 2 lines of vision was observed in the group with lower baseline visual acuity (<54 letters). However, none of the participants with lower baseline vision reached driving vision. Participants with better baseline vision (≥54 letters) achieved better final visual acuity with 38% achieving driving standard BCVA at 24 months.

**Table 2:** Outcomes measures in terms of visual acuity and anatomical changes at 12 months from baseline.

	All patients N = 34	VA <54 lettersN = 10	VA ≥54 letters N = 24	CMT <400 µm N = 12$	CMT ≥400 µm N = 22
Baseline median VA (range)	58.5 (42–69)	47.5 (42–53)	60.0 (54–69)	61.0 (53–69)	57.0 (42–69)
Median change in VA (range)	8.0 (−18 to 16)	8.5 (−3 to 16)	7.0 (−18 to 15)	7.0 (−10 to 13)	8 (−18 to 16)
Median final visual acuity(range)	63.5 (44–79)	57.5 (44–68)	67.0 (47–79)*	67.0 (47–78)	62.0 (44–79)
Proportion with final visualacuity >73 letters	3 (8.8)	0 (0)	3 (14.3)	1 (8.3)	2 (9.1)
Proportion with ≥10 lettergain	11 (32.4)	4 (30.8)	7 (33.3)	3 (25.0)	8 (36.4)
Proportion with <10 lettergain	23 (67.6)	6 (60.0)	17 (70.8)	9 (25.0)	14 (63.6)
Proportion with ≥15 lettergain	3 (8.8)	2 (20.0)	1 (4.2)	0 (0)	3 (13.6)
Proportion with <15 lettergain	31 (91.2)	8 (80.0)	23 (95.8)	12 (100.0)	19 (86.4)
Proportion with ≥30 lettergain	0 (0)	0 (0)	0 (0)	0 (0)	0 (0)
Proportion with <30 lettergain	34 (100)	10 (100.0)	24 (100.0)	12 (100.0)	22 (100.0)
Proportion with ≥10 letterloss	1 (2.9)	0 (0)	1 (4.2)	0 (0)	1 (4.5)
Proportion with ≥15 letterloss	1 (2.9)	0 (0)	1 (4.2)	0 (0)	1 (4.5)
Proportion with≥30 letterloss	0 (0)	0 (0)	0 (0)	0 (0)	0 (0)
Median change in CMT(range)	−106.5 (−496 to 92)	−182.5 (−496 to 18)	−86.5 (−407 to 92)	−50.5 (−187 to 20)	−**137.5 (**−496 to 92)*
Median final CMT(range)	374.0 (167–663)	436.0 (215–663)	321.0 (167–617)*	286.5 (167–405)	430.0 (173–663)*
Proportion with final CMT<250 µm	6 (17.6)	1 (11.1)	5 (20.8)	3 (25.0)	3(14.3)
Median % change in excessretinal thickness	−43.3	−36.5	−43.3	−29.7	−43.3
Proportion of patients with 2step reduction of logOCTn(%)	8(25)	3(33.3)	5 (21.7)	3(27.3)	5(23.8)
Median number of injectionsin 12 months (range)	9.0 (5–9)	9.0 (8–9)	8.5 (5–9)	8.5 (5–9)	9.0 (5–9)

BOLD = significant p<0.05;

$ 1 missing value; VA-visual acuity; SD- standard deviation; CMT –central macular thickness.

**Table 3:** Visual acuity and anatomical outcome measures at 24 months compared to baseline.

	All patients N = 34	VA <54 lettersN = 10	VA ≥54 letters N = 24	CMT <400 µmN = 12	CMT ≥400 µm N = 22
Median change in VA (range)	9.5 (−9 to 28)	9.0 (−9 to 24)	10.5 (−9 to 28)	8.5 (−9 to 24)	9.5 (−9 to 28)
Median final visual acuity (range	67.0 (38–88)	55.5 (38–77)	68.0 (48–88)*	68.0 (48–83)	66.5 (38–88)
Proportion with final visual acuity >73 letters	9 (26.5)	1 (10.0)	8 (33.3)	4(33.3)	5(22.7)
Proportion with ≥10 letter gain	17 (50.0)	5 (50.0)	12 (50.0)	6(50.0)	11(50.0)
Proportion with <10 letter gain	17 (50.0)	5 (50.0)	12 (50.0)	6(50.0)	11(50.0)
Proportion with ≥15 letter gain	11 (32.4)	4 (40.0)	7 (29.2)	4 (33.3)	7 (31.8)
Proportion with <15 letter gain	23 (67.6)	6 (60.0)	17 (70.8)	8 (66.7)	15 (68.2)
Proportion with ≥30 letter gain	0 (0)	0 (0)	0 (0)	0 (0)	0 (0)
Proportion with <30 letter gain	34 (100.0)	10 (100.0)	24 (100.0)	12 (100.0)	22 (100.0)
Proportion with ≥10 letter loss	0 (0)	0 (0)	0 (0)	0 (0)	0 (0)
Proportion with ≥15 letter loss	0 (0)	0 (0)	0 (0)	0 (0)	0 (0)
Proportion with≥30 letter loss	0 (0)	0 (0)	0 (0)	0 (0)	0 (0)
Median % reduction in visualacuity deficit	36.0	24.0	41.67	34.7	37.7
Median change in CMT (range)	−121.0 (−477 to 177)	−131.5 (−438 to 103)	−122.5 (−477 to 123)	−94.0 (−160 to 54)	−169.5 (−477 to 123)
Median final CMT (range)	340.5 (117–852)	381.0 (220–852)	292.0 (117–645)	251.5 (156–−439)	381.0 (117 to 852) ns
Proportion with final CMT<250 µm	12 (35.3)	2 (20.0)	10 (41.7)	6(50.0)	6(27.3)
Mean % reduction in CMT	−56.0	−39.9	−58.8	−68.1	−49.3
Proportion of patients with 2 stepreduction of logOCT	15(45.5)	5 (50.0)	10 (43.5)	5(41.7)	10(47.6)
Median number of injections inyear 2 (range)	5.0 (0–9)	5.5 (4–7)	4.0 (0–9)	4.5 (0–9)	5.0 (0–7)

**BOLD = significant p<0.05** VA-visual acuity; SD- standard deviation; CMT –central macular thickness.

### Change in Central Macular Thickness

The mean reduction in CMT of the cohort was approximately 121 (SD 117) µm at 24 months and was independent of baseline BCVA. Seventy-nine percent still had centre involving macular oedema at 12 months, decreasing to 65% at 24 months. The proportion of participants with a CMT≥300 µm at 12 and 24 months were 65% and 56% respectively. Participants with a CMT≥400 µm remained significantly thicker at 12 and 24 months, than those with CMT<400 µm. The mean percentage reduction in excess macular thickness was comparable in the two categories of baseline macular thickness (300 and 400 µm) at both time-points (Tables 2 and 3).

There was no significant correlation between baseline visual acuity or baseline CMT with change in visual acuity or change in macular thickness. Thirty percent of the participants achieved the aforementioned composite score of a gain of 10 or more letters associated with a >20% reduction of CMT, at month 12 and 24.

### Patterns of Evolution of Macular Oedema Over Time

The findings are summarised in Table 4. At 12 months, 30% of the participants responded after 3 loading injections and maintained that effect to 12 months (‘Early and consistent’ pattern), associated with the best visual outcome compared to other patterns of response. However, half of these participants then failed to maintain this effect at 24 months.

**Table 4:** The evolution patterns of diabetic macular oedema at 12 and 24 months.

Patterns of evolution	No: of Eyes	Median change in VA (IQR) at 12 months	Response at 24 months		
			Sustained responders	stabilisers	Late Poor responders
Early and consistent	10	10.5 (4 to 16)	2	3	5
Early and inconsistent	2	8.5 (8 to 9)	1	0	1
Slow and variable	7	1.0 (−18 to 13)	1	4	2
Non responder	3	9.0 (2 to 13)	2	0	1

### Associations between Baseline Characteristics and Change in Visual Acuity and Central Macular Thickness

Table 5 shows the fully adjusted effect size and confidence intervals of associations between baseline characteristics and change in visual acuity and macular thickness at 24 months. We identified that better baseline visual acuity was associated with a better visual acuity at both 12 and 24 months, but did not influence the change in visual acuity.

**Table 5:** Associations between baseline covariates and visual acuity and macular thickness at 2 years.

	Change in final Visual Acuity (24-b)*		N = 34	Change in final Macular Thickness (24-b)**		N = 34
Baseline covariates	Effect size (η_p_^2^)	95% CI	P-value	Effect size (η_p_^2^)	95% CI	P-value
Age	0.001	−0.53, 0.61	0.88	0.029	−5.82, 11.48	0.50
Gender	0.011	−15.25, 10.16	0.68	0.150	−39.74, 346.04	0.112
Baseline HbA1C	0.002	−0.003, 0.002	0.88	0.006	−0.05, 0.04	0.766
Baseline visual acuity	0.014	−1.17, 0.74	0.64	0.035	−19.66, 9.32	0.46
Duration of diabetes	0.000	−0.05, 0.06	0.94	0.003	−0.72, 0.88	0.84
Duration of CSMO	0.079	−0.39, 0.11	0.26	0.003	−4.19, 3.46	0.84
Number of previous laser treatments	0.02	−0.1.79, 2.98	0.60	0.101	−59.03, 13.37	0.20
Macular thickness	0.027	−0.07, 0.04	0.511	0.087	−1.32, 0.35	0.234
Area of foveal avascular zone	0.03	−8.34 16.72	0.489	0.000	−189.87, 190.62	0.997
Diameter of fovealavascular zone	0.006	−0.02, 0.02	0.76	0.036	−0.44, 0.20	0.451
Diameter of fovealavascular zone	0.006	−0.02, 0.02	0.76	0.036	−0.44, 0.20	0.451

Where *R^2^ = 0.184, and **R^2^ = 0.286.

## Discussion

This exploratory investigation of the BOLT study provides detailed descriptive analyses of the changes in visual acuity and central macular thickness and predictive factors of response of DMO to bevacizumab over 24 months. We highlight here that all bevacizumab trials to date are of relatively small sample size compared to the ranibizumab trials thereby making comparison challenging. However, as bevacizumab is by far the most commonly used antiVEGF agent globally, it is important that this randomised controlled trial of bevacizumab in DMO with 24 months follow-up be analysed in the format used in the ranibizumab studies [7], [8] to better inform clinicians, patients and funders, rather than solely make assumptions that the drugs are similar for treating DMO based on published trials comparing the 2 drugs in age related macular degeneration.

The overall functional outcomes of the bevacizumab arm of the BOLT study at 12 and 24 months are similar to that of the ranibizumab studies [4], [7], [8] despite using a 6 weekly interval loading phase and different re-treatment criteria. Nevertheless the re-treatment criteria in the BOLT, RESTORE and DRCR.net studies were designed on the same principle of initiation phase followed by individualised PRN dosing schedule based on disease progression as judged by visual acuity and OCT changes, with a low threshold for re-treatment. In the RESTORE study, from month 3 to 11, monthly injections were given until vision was stabilised (i.e. at the 2 last consecutive visits no BCVA improvement observed due to ranibizumab or BCVA >84 letters). Injections were re-initiated if there was BCVA decrease due to DMO progression. In DRCR.net study, re-injection was done in all visits in which there was an evidence of improvement. If success or failure criteria were met it was left to the investigator’s discretion to re-inject. The visual acuity eligibility criterion of the BOLT study was also different, with a lower visual acuity range of 35–69 letters, unlike the DRCR.net study where 50% of the study eyes had ≥66 letters, and 19.8% of the RESTORE patients who a BCVA of more than 73 letters.

Despite these aforementioned differences in baseline characteristics, the patterns of response of DMO to ranibizumab and bevacizumab are very similar when standard outcomes are assessed. But, this study also highlights important differences that should be explored in adequately powered larger studies. The median baseline CMT in the bevacizumab arm in the BOLT study for patients who exited at 24 months was higher than that of patients who completed 2 years in the DRCR.net trial, suggesting that comparisons between the drugs should be made with caution. However, whilst in the ranibizumab studies [4], [7], [8] approximately 40% had persistent macular oedema defined as OCT central subfield ≥250 µm and only 20%–24% had more than mildly thickened retina defined as OCT central subfield ≤250 µm at two years, the corresponding figures in the bevacizumab arm of the BOLT study were 65% and 56% respectively. This suggests that further studies should explore the concept that the ‘drying effect’ of bevacizumab may be less effective than for Ranibizumab [16].

Despite the fact that greater reductions in thickness were observed in BOLT participants with macular thickness of ≥400 µm compared to those with ≤400 µm, the mean macular thickness was still ≥400 µm at 12 and 24 months, and the proportion that achieved a dry macula (CMT<250 µm) was limited to 14% and 27% at 12 and 24 months respectively. Similar effects were observed in the DRCR.net study. However, the proportion with ≥2 step log improvement in LogOCT was lower in the BOLT study compared to the DRCR.net study. Although the BOLT study is a small study, these exploratory analyses provide support for a comparative study of ranibizumab and bevacizumab.

Some of these differences may be related to differences in molecular weight, half-life and degree of retinal penetration of bevacizumab and ranibizumab based on their structural differences. However, the importance of these proposed differences is controversial [17], [18], [19], [20]. Further studies are required to elicit the optimal therapeutic effect of bevacizumab in different retinal thickness profiles.

Another noteworthy finding was that eyes with rapid and consistent response to bevacizumab had the best outcome at 12 months compared to all the other OCT patterns of resolution of oedema. This is in keeping with the ranibizumab studies [7], [8]. However, an increase in macular thickness of at least 20% was observed in 50% of these participants by 24 months. This observation mirrors the CATT study, in which persistent fluid at the macula was more frequently observed in the bevacizumab arm compared to Ranibizumab [14]. This delayed non-response or rebound phenomenon also merit further evaluation in future studies.

The inherent limitation of this study is the small sample size. Nevertheless, the trends shown in these exploratory analyses justify further investigation, since they have important implications on the potential choice of anti-VEGF agent to use in eyes with DMO. Another limitation is the use of Stratus OCT, a time domain OCT which is subject to frequent artefacts and lower repeatability compared to spectral-domain OCT. Further sufficiently powered clinical trials comparing various antiVEGF agents with at least 24 months follow-up and using spectral domain OCT will better inform clinicians on subtle differences in the effects on macular thickness profiles.
