# Correction: Structural and Functional Measures of Efficacy in Response to Bevacizumab Monotherapy in Diabetic Macular Oedema: Exploratory Analyses of the BOLT Study (Report 4)

**DOI:** 10.1371/annotation/a9d300ac-887d-43b3-a9b1-425d33cdf13b

**Published:** 2013-09-04

**Authors:** Sobha Sivaprasad, Roxanne Crosby-Nwaobi, Simona Degli Esposti, Tunde Peto, Ranjan Rajendram, Michel Michaelides, Philip Hykin

There was an error in the name of the third author.

The correct name is: Simona Degli Esposti

The correct citation is: Sivaprasad S, Crosby-Nwaobi R, Esposti SD, Peto T, Rajendram R, et al. (2013) Structural and Functional Measures of Efficacy in Response to Bevacizumab Monotherapy in Diabetic Macular Oedema: Exploratory Analyses of the BOLT Study (Report 4). PLoS ONE 8(8): e72755. doi:10.1371/journal.pone.0072755

The correct Author Contributions are: Conceived and designed the experiments: SS. Performed the experiments: SDE TP RR MM. Analyzed the data: RCN. Contributed reagents/materials/analysis tools: RCN TP. Wrote the paper: SS MM PH. 

